# Microbiological and Clinical Aspects of *Actinomyces* Infections: What Have We Learned?

**DOI:** 10.3390/antibiotics10020151

**Published:** 2021-02-03

**Authors:** Edit Urbán, Márió Gajdács

**Affiliations:** 1 Department of Medical Microbiology and Immunology, University of Pécs Medical School, Szigeti út 12., 7624 Pécs, Hungary; zsoldiedit25@gmail.com; 2 Institute of Translational Medicine, University of Pécs Medical School, Szigeti út 12., 7624 Pécs, Hungary; 3 Department of Pharmacodynamics and Biopharmacy, Faculty of Pharmacy, University of Szeged, Eötvös utca 6., 6720 Szeged, Hungary; 4 Institute of Medical Microbiology, Faculty of Medicine, Semmelweis University, Nagyvárad tér 4., 1089 Budapest, Hungary

Obligate anaerobic bacteria are important members of the normal human microbiota, present in high numbers on mucosal surfaces (e.g., the oral cavity, female genital tract, and colon), outnumbering other bacteria 10–1000-fold [1]. Anaerobic bacteria have been implicated in a wide range of infectious processes from almost all anatomical sites, by bacteria from both exogenous (e.g., toxin-mediated pathologies by Clostridia) and endogenous (displacement of the bacterial flora to other anatomical regions) sources [2]. These pathogens may be important etiological agents in life-threatening, invasive infections [3,4]. The cultivation and identification of strict anaerobes is labor-intensive and requires expertise and special laboratory conditions and equipment; therefore, for many years, only several anaerobes were considered clinically relevant [5]. With the emergence and spread of modern identification technologies—such as polymerase chain reaction (PCR), matrix-assisted laser desorption/ionization time-of-flight mass spectrometry (MALDI-TOF MS), and 16S RNA gene sequencing—in clinical microbiology laboratories, the pathogenic role of these microorganisms may be established more realistically [6]. 

Non-spore-forming Gram-positive anaerobes include the genera *Actinomyces*, *Arachnia*, *Bifidobacterum*, *Bryantella*, *Cutibacterium* (previously *Propionibacterium*), and *Eubacterium* [7]. The genus name “*Actinomyces*” originates from the Greek words “*aktina*” («ακτίνα») (ray) and “*mykis*” («μύκης») (fungus), which aims to symbolize the arrangement of the bacterial filaments, which may frequently be observed with microscopy [8]. As of now, there have been 26 individual *Actinomyces* species implicated in human clinical infections (actinomycoses), with *A. odontolyticus*, *A. meyeri*, *A. israelii*, and *A. gerencseriae* responsible for more than 90% of these infections [9,10]. Most cases (40–60%) of actinomycoses affect the cervicofacial region and the central nervous system (CNS), while abdominal (20–30%), thoracic/pulmonary (20–30%), pelvic (3–5%), and cutaneous (3–5%) manifestations must also be considered [9,10,11,12,13]. The clinical diagnosis of actinomycosis may be quite difficult: the clinical presentation of the patient, past medical history (injuries, immunosuppression, cancers), laboratory parameters, microbiological culture, and histopathology must all be taken into consideration, as this disease frequently mimics a malignancy or infection by other bacteria (e.g., *Nocardia*, mycobacteria) [11,12,13]. Nonetheless, successful culture is also hindered by the slow growth rate and fastidious growth requirements of *Actinomyces* species [6,9]. 

Although actinomycoses are thought to be rarely occurring infections, there is very limited data available on their epidemiology: the prevalence of actinomycoses has been estimated to be around 1/300,000 persons, but this estimation is largely outdated [14]. This may be due to, among other things, the fact that actinomycoses are not included in the World Health Organization (WHO) Recommended Surveillance Standards or in the national bacteriological surveillance of many countries. As up-to-date references were scarce on the topic of *Actinomyces* infections, the Special Issue “Microbiological and Clinical Aspects of Actinomyces Infections” was established with the aim of enriching the existing literature regarding these neglected pathogens with original articles, case reports, and comprehensive review papers. In the following section, we would like to present a short summary of the papers published in our Special Issue to prompt further interest in these articles.

Gajdács et al. performed a much-needed summary of the currently available literature on the pathogenic role of *Actinomyces* species and *Actinomyces*-like organisms (ALOs) in the female and male genital tract (genital tract actinomycosis is the second most common manifestation of the disease) and in urinary tract infections (UTIs), in addition to infections associated with intrauterine devices (IUDs). The review also contained a separate section concerning the antibiotic therapy and antibiotic resistance (including the production of *bla*_TEM_-type β-lactamases and reduced membrane permeability) of *Actinomyces* species and ALOs [15]. 

Džupová et al. described the case of a 35-year-old female patient with disseminated pelvic actinomycosis secondary to an IUD [16]. The patient postponed seeking medical attention due to fears of repercussions in the workplace and fears of a tumor diagnosis. After a battery of laboratory tests (which did not confirm the presence of immunosuppression or HIV), computer tomography (CT), and a biopsy, the diagnosis of metastatic ovarian cancer was assumed; in addition, bilateral ureteral stricture was also detected. Interestingly, the involvement of actinomycosis (*A. naeslundii*) was only revealed after the examination of a liver biopsy sample. In addition, the principal symptoms of the patient were prolonged exhaustion, malaise, and cachexia, while the signs on the affected tissues were only noticeable much later on. The patient underwent extensive surgery, the IUD was removed, and she received antibiotic therapy for nine months. This case highlights the difficulties of differential diagnosis (actinomycosis vs. malignancy), in addition to the renal involvement, which is frequently noted in pelvic actinomycosis [16].

Chaves et al. described a case of pulmonary actinomycosis in a 35-year-old female with bronchopulmonary sequestration, who had complaints of dyspnea and persistent coughing and a 4-year history of recurrent pneumonia [17]. During the clinical workup, a CT scan of the chest revealed a multicystic mass vascularized by an aberrant vessel in the left lung of the patient, which prompted the clinicians to perform surgery. Just before the procedure, the patient had a high white blood cell (WBC) count with neutrophil dominance. During the surgery, a lung fragment was extracted, which was sent for histopathological examination: here, filamentous nonsporulating bacilli were seen, which were identified as *Actinomyces* spp. (via histological staining, sulfur granules). Interestingly, as the patient was asymptomatic at the time and the laboratory parameters were close to the normal range, no antibiotic therapy was ordered [17]. 

D’Amore et al. presented a case of lingual actinomycosis in a 52-year-old male patient, complaining of a recent, asymptomatic, nonulcerated swelling of the anterior part of the tongue [18]. The patient reportedly had a benign squamous papilloma on the same location of this tongue 5 years prior. Laboratory tests indicated neutropenia, monocytosis, and elevated C-reactive protein (CRP) levels. During the biopsy (for histopathological diagnosis), a yellowish purulent discharge was noted. This, together with the histological examination of the sample, led to the diagnosis of actinomycosis of the tongue. The patient received clarithromycin for seven days, as he had a known penicillin allergy. Overall, the patient was complaint-free after the short course of antibiotics [18].

Stájer et al. described two distinct clinical cases of cervicofacial actinomyces infections with the antibiotic susceptibilities of the implicated pathogens; in addition, the authors provided an epidemiological summary of cervicofacial infections in their institution between 2005 and 2015 [19]. The first case was from a 22-year-old female patient who was of high social status and had generally good oral and personal hygiene; the patient had no known underlying conditions except for penicillin allergy. Based on the clinical presentation, the presumptive diagnosis of cervicofacial actinomyces was established, and samples were sent to the microbiology department. The samples were positive for *A. israelii*, *Clostridium ramosum*, *C. clostridioforme*, *Prevotella bivia* (strict anaerobes), *Capnocytophaga* spp., and *Eikenella corrodens* (facultative anaerobes). The patient received clindamycin and metronidazole for seven days, with instructions to take clindamycin for an additional seven days. In contrast, the authors described a second, more complicated case of a 40-year-old patient from a low socioeconomic standing with neglected oral hygiene, following a traumatic injury. Following surgical intervention, the patient received amoxicillin, but the adherence of the patient was unsatisfactory. Six months later, the patient returned with pain and swelling in the area of the previous injury and partial trismus. After an incision was made, a sample for culture was sent to the microbiology laboratory, which reported *A. israelii* in high colony counts. The infected surgical site was drained, and the patient received amoxicillin intravenously and later per os. The highlight of the present article is that cervicofacial actinomyces infections are rare, but they must be considered in patients from all different backgrounds and medical histories [19]. 
